# Racial and Socioeconomic Disparities in Sudden Unexpected Infant Death in the United States: A Population-Based Analysis Using Linked Birth-Infant Death Data

**DOI:** 10.7759/cureus.106686

**Published:** 2026-04-08

**Authors:** Onize Ekome, Akinyele Oladimeji, Chetachukwu G Amalu, Esther M Adjei, Saare Abera

**Affiliations:** 1 Mental Health, Braxia Health, Mississauga, CAN; 2 Family Medicine, Alberta Health Services, Edmonton, CAN; 3 Internal Medicine, Ebonyi State University Teaching Hospital, Abakaliki, NGA; 4 Internal Medicine, The Trust Hospital Company Limited, Accra, GHA; 5 Internal Medicine, American University of Integrated Sciences, St. Michaels, BRB

**Keywords:** birth weight, gestational age, health disparities, infant mortality, maternal education, sudden unexpected infant death

## Abstract

Background: Sudden unexpected infant death (SUID) remains an important contributor to infant mortality in the United States. Previous national surveillance reports have documented differences in mortality across racial, socioeconomic, and birth-related characteristics. Continued examination of these patterns using national vital statistics data is important for understanding population-level disparities and informing prevention strategies.

Objective: To quantify disparities in SUID in the United States through 2023 by examining social determinants, including race, ethnicity, and maternal education, alongside clinical characteristics such as birth weight and gestational age.

Methods: A descriptive cross-sectional study was conducted using the Linked Birth Infant Death dataset accessed through the CDC Wide-Ranging Online Data for Epidemiologic Research system. The study included aggregated national data from 2007 to 2023, representing 66,381,536 live births and 61,594 SUIDs. Maternal age, maternal education, maternal race and ethnicity, birth weight, and gestational age were examined. Mortality rates were calculated as deaths per 1,000 live births within each category.

Results: Higher mortality rates were observed among infants born to younger mothers and mothers with lower educational attainment. Mortality also varied across racial and ethnic groups, with higher rates among infants born to non-Hispanic Black mothers. Birth-related characteristics showed similar patterns, with higher rates among infants with low birth weight and earlier gestational age.

Conclusion: This study highlights persistent disparities in SUID across maternal demographic and birth-related characteristics in the United States. Continued public health efforts focused on safe sleep education, maternal health, and targeted prevention strategies may help address these disparities.

## Introduction

Sudden unexpected infant death (SUID) is still among the main causes of death among infants in the United States [1]. Despite a record low in overall infant mortality in the United States in 2020, the rate of SUID increased relative to 2019 [2]. It includes multiple types of infant mortality, such as sudden infant death syndrome (SIDS), accidental suffocation and strangulation in bed, and other ill-defined or unknown causes of mortality [3]. Some of the aspects that have been observed as preventive to SUIDs are supine position during sleep, the use of a firm sleep surface, breastfeeding, provision of pacifier, sleeping with any other person, the removal of any soft object in the bed or beneath the infant, prevention of overheating, and the use of tobacco, alcohol, and illicit drugs [4,5]. Although the infant’s mortality level in general is declining with the years, SUID has remained a major contributor to infant mortality, which can be avoided; thus, additional investigations into its determinants and risk factors are required [6].

Over the past two decades, a number of public health programs have been introduced in the fight against SUID [7]. Since the late 1980s, Back-to-Sleep (BTS) campaigns have been conducted in most developed nations to raise awareness of the protective effect of the supine position in case of infant death during sleep [8,9]. Caregiver receipts of, and interaction with, public health messages, along with recognizing trends in modern infant care practices, are equally important elements that are necessary to aptly inform and clarify future safe sleeping guidance [10]. Increasing the adoption of safe sleep messages by families with young infants, particularly those at increased risk, has the potential to avert infant mortality [11].

Studies have shown that infant mortality is one of the vital parameters of population health since the health and survival of infants are predetermined by the features of society where they have to be born [12]. Similar to racial/ethnic differences in maternal mortality, Black women are prone to severe maternal morbidity compared to their White counterparts [13]. On a national level, infants born as non-Hispanic Black (NHB) are twice as likely and those born Hispanic a bit more than that to die of SUD as compared to non-Hispanic White (NHW) infants [14]. A number of other risk factors have also been pinpointed to be linked to SIDS, among others: extrinsic factors, such as cigarette smoke exposure, alcohol and drug use, and bottle feeding, and intrinsic factors, such as demography, gender, pre-term births, and intrauterine pathology [15]. 

There is also the importance of socioeconomic factors in determining infant health outcomes. The level of maternal education, family income, prenatal care availability, and living standards could each contribute to the threat of infant mortality [16]. Socioeconomically disadvantaged families can have problems with access to sufficient prenatal and postnatal care, and reduced access to safe housing and health education resources [17]. These circumstances can raise the risk of exposure to the risk factors of unsafe sleep habits, maternal health complications, or environmental risks that cause SUIDs [18].

The study will utilize CDC WONDER and Linked Birth-Infant Death Data to offer better analysis. The Wide-Ranging Online Data for Epidemiologic Research (WONDER) database of the Centers for Disease Control and Prevention (CDC) provides comprehensive data and information regarding infant death data in the United States [19]. Similarly, Linked Birth-Infant Death Data provides comprehensive data files on population-based infant death data of the United States [20]. The objective of this study is to quantify disparities in SUID in the United States through 2023 by examining social determinants, including race, ethnicity, and maternal education, alongside clinical characteristics such as birth weight and gestational age. The results of the study will offer useful insights into the developing information regarding the effectiveness of community health in reducing infant mortality.

## Materials and methods

Study design and data source

This study used a descriptive cross-sectional design to examine patterns of SUID in the United States. Data were obtained from the Linked Birth Infant Death dataset available through the CDC Wide-Ranging Online Data for Epidemiologic Research system [21]. This dataset links infant death records to corresponding birth certificates within the national vital statistics system and provides population-level information on maternal characteristics, birth outcomes, and causes of infant death. The dataset includes births and infant deaths occurring among United States residents and is compiled by the National Center for Health Statistics. For this study, data from 2007 through 2023 were analyzed to evaluate disparities in SUID across maternal demographic and birth-related characteristics. Temporal trends over the study period were not examined, as the analysis focused on overall patterns using aggregated data.

Study population

The study population included all live births recorded in the United States during the study period that were available within the linked birth infant death dataset. Infant deaths classified as SUID were identified using the underlying cause of death codes recorded in the mortality data. SUID was defined using International Classification of Diseases, Tenth Revision (ICD-10) codes (including SIDS, accidental suffocation and strangulation in bed, and other ill-defined causes) as classified within the Centers for Disease Control and Prevention Linked Birth-Infant Death dataset, which follows standardized coding by the World Health Organization. The final analytic dataset consisted of aggregated counts representing 61,594 SUIDs among 66,381,536 live births, categorized by maternal age, maternal education, maternal race and ethnicity, birth weight, and gestational age.

Variables and measures

The primary outcome was SUID, defined according to the ICD 10 cause of death codes included in the linked dataset. Maternal demographic characteristics included maternal age and maternal educational attainment. Maternal age was grouped into the categories younger than 20 years, 20-24 years, 25-29 years, 30-34 years, 35-39 years, and 40 years or older. Maternal education was categorized as less than high school, high school graduate, some college, associate degree, bachelor's degree, and graduate degree. Maternal education was used as the primary socioeconomic indicator because it is the only consistently available socioeconomic variable within the aggregated Linked Birth-Infant Death dataset accessed through CDC WONDER.

Birth-related characteristics included infant birth weight and gestational age. Birth weight was classified as less than 1500 grams, 1500-2499 grams, 2500-3499 grams, 3500-3999 grams, and 4000 grams or greater. Gestational age was grouped as less than 28 weeks, 28-31 weeks, 32-36 weeks, 37-41 weeks, and 42 weeks or more. For each category, the number of SUID deaths, the number of live births, and the mortality rate per 1,000 live births were calculated.

Missing data

The linked birth infant death dataset contains national vital statistics data derived from standardized birth and death certificates. Some variables may contain missing or incomplete information due to incomplete reporting on the original certificates. In this study, analyses were conducted using aggregated counts provided within the CDC WONDER query system, which does not allow assessment of the extent or patterns of missing data at the individual level. Categories with missing or unspecified information were retained when present in the source data, although the primary analysis focused on reported categories of maternal and birth characteristics. Because the analysis used aggregated data, individual-level imputation procedures were not performed.

Statistical analysis

Descriptive analyses were conducted to examine the distribution of SUID across maternal demographic and birth-related characteristics. For each category of maternal age, maternal education, birth weight, gestational age, and maternal race and ethnicity, the number of SUID deaths and the number of live births were obtained from the CDC WONDER query system. Mortality rates were calculated as the number of SUID deaths per 1,000 live births within each category. The results were presented using summary tables and graphical displays to illustrate differences in mortality rates across groups, and measures of statistical uncertainty, such as confidence intervals or hypothesis testing, were not performed. All data processing, organization of aggregated counts, and generation of tables and figures were performed using Stata version 18 [22].

Ethical considerations

This study used publicly available aggregated data obtained from the CDC WONDER system. The dataset does not contain individual-level identifiers or personal information that could be used to identify specific individuals. Because the analysis relied on deidentified national vital statistics data that are publicly accessible, institutional review board approval was not required. The study followed principles for the responsible use of publicly available health data and complied with the data use guidelines provided for the CDC WONDER system.

## Results

Table 1 presents the distribution of SUID across selected maternal and birth characteristics in the United States during the study period. The table summarizes the number of SUID deaths, the number of live births, and the corresponding mortality rates per 1,000 live births across categories of maternal age, maternal education, birth weight, and gestational age.

**Table 1:** Maternal and Birth Characteristics Associated With SUID in the United States, 2007-2023 (N = 61,594 SUID deaths among 66,381,536 live births) SUID refers to sudden unexpected infant death. Rates are expressed as deaths per 1,000 live births. Data derived from the Linked Birth-Infant Death dataset (2007-2023) accessed via CDC WONDER. The data used in this analysis consist of aggregated national counts and rates. Statistical processing and table preparation were conducted using Stata version 18 [22]. SUID, sudden unexpected infant death.

Characteristic	SUID Deaths	Live Births	Rate per 1,000
Maternal Age			
<20 years	8,551	4,442,135	1.92
20-24 years	21,845	14,133,056	1.55
25-29 years	16,767	18,873,314	0.89
30-34 years	9,611	17,833,661	0.54
35-39 years	3,976	8,993,686	0.44
≥40 years	841	2,091,989	0.40
Maternal Education			
Less than high school	13,223	8,413,449	1.57
High school graduate	19,974	14,486,986	1.38
Some college	11,472	11,208,691	1.02
Associate degree	2,532	4,462,570	0.57
Bachelor's degree	2,846	10,988,171	0.26
Graduate degree	1,112	6,347,781	0.18
Birth Weight			
<1500 grams	2,802	951,104	2.95
1500-2499 grams	10,282	4,521,735	2.27
2500-3499 grams	36,059	38,289,617	0.94
3500-3999 grams	9,934	17,505,301	0.57
≥4000 g	2,428	5,090,799	0.48
Gestational Age			
<28 weeks	553	222,508	2.49
28-31 weeks	959	311,008	3.08
32-36 weeks	5,616	2,885,968	1.95
37-41 weeks	24,530	30,316,978	0.81
≥42 weeks	77	99,541	0.77

The results indicate variation in SUID mortality across maternal age groups. The highest rate was observed among infants born to mothers younger than 20 years, 1.92 deaths per 1,000 live births, while the lowest rate occurred among mothers aged 40 years or older, 0.40 deaths per 1,000 live births. Mortality rates declined progressively with increasing maternal age. Differences were also observed across maternal education categories. Infants born to mothers with less than a high school education had a rate of 1.57 deaths per 1,000 live births, while those born to mothers with graduate-level education had a rate of 0.18 deaths per 1,000 live births. The rates decreased steadily with higher levels of maternal education. Patterns were also evident across birth weight categories. Infants weighing less than 1500 grams had a rate of 2.95 deaths per 1,000 live births, whereas infants weighing 4000 grams or more had a rate of 0.48 deaths per 1,000 live births. Mortality rates were lower among infants in the higher birth weight categories. Similar variation appeared across gestational age groups. Infants born at 28 to 31 weeks of gestation had the highest rate, 3.08 deaths per 1,000 live births, while infants born at 37 to 41 weeks had a rate of 0.81 deaths per 1,000 live births. Overall, the results show that SUID mortality rates differed across maternal demographic characteristics and birth-related factors in this national dataset.

**Figure 1:**
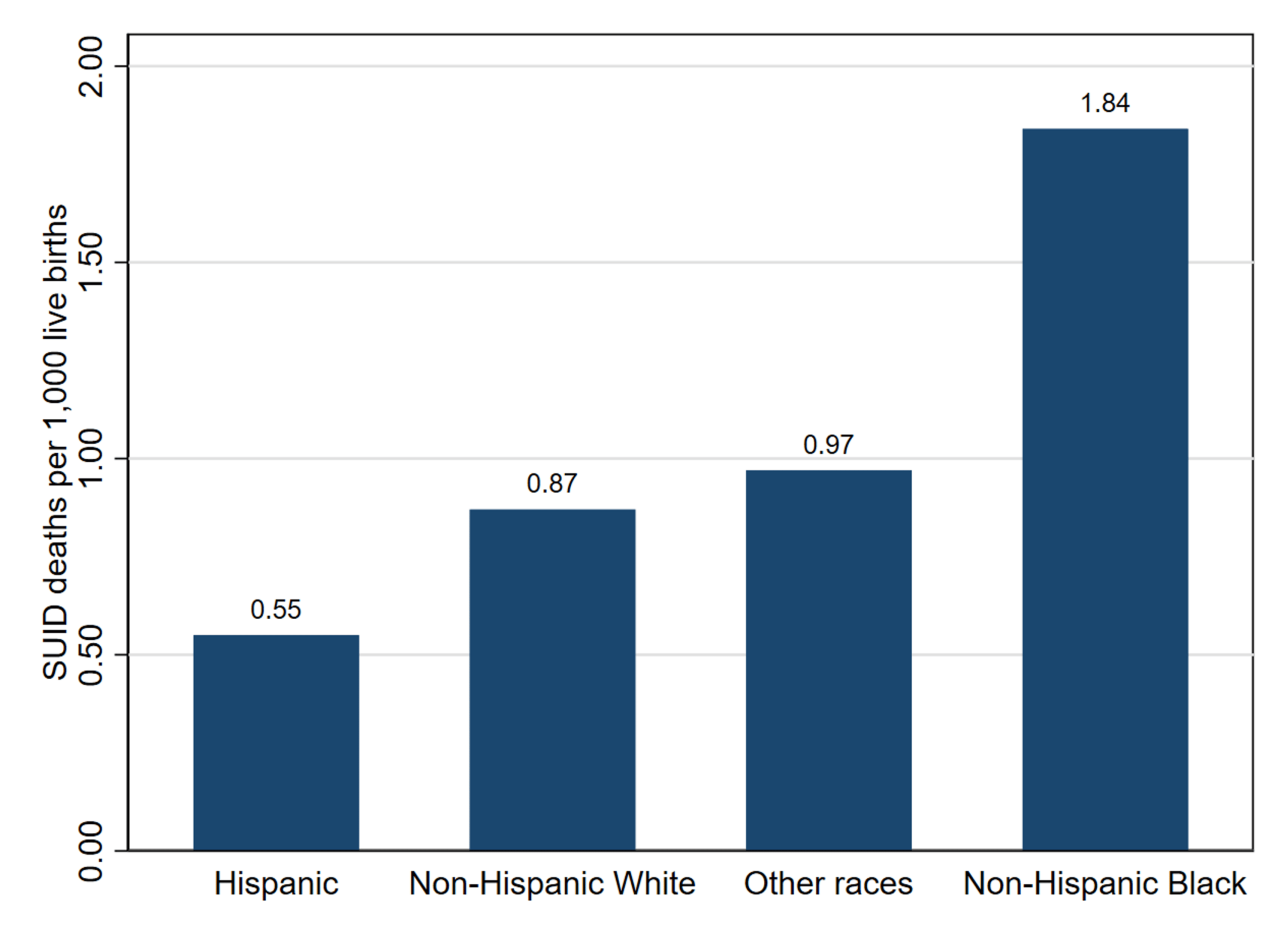
SUID Mortality Rates by Maternal Race and Ethnicity in the United States, 2007 to 2023. SUID, sudden unexpected infant death.

Figure 1 illustrates the variation in SUID mortality rates across maternal race and ethnicity categories. The figure presents mortality rates per 1,000 live births among Hispanic, NHW, other racial groups, and NHB populations.

The figure shows differences in SUID mortality rates across racial and ethnic groups. Infants born to NHB mothers had the highest mortality rate, approximately 1.84 deaths per 1,000 live births. Lower rates were observed among NHW infants, about 0.87 deaths per 1,000 live births. Rates among other racial groups were approximately 0.97 deaths per 1,000 live births, while Hispanic infants had the lowest rate at approximately 0.55 deaths per 1,000 live births. The results indicate that SUID mortality rates varied across maternal racial and ethnic groups in the study population.

## Discussion

This study focuses on the distribution of SUID across maternal and birth characteristics in the United States using nationally aggregated data. The findings show clear variation in mortality rates across maternal age, maternal education, birth weight, gestational age, and maternal race and ethnicity. Infants born to younger mothers had higher mortality rates compared with infants born to older mothers. Mortality also decreased as maternal education increased. Differences were also observed across birth characteristics. Infants with lower birth weight and those born at earlier gestational ages had higher mortality rates compared with infants with higher birth weight and those born at term. In addition, the graphical analysis demonstrated variation in mortality across racial and ethnic groups, with higher rates among infants born to NHB mothers compared with other groups. These findings reflect patterns reported in national surveillance studies of SUIDs in the United States [1,6]. Prior analyses using national datasets have documented persistent differences in SUID mortality across racial and socioeconomic groups, and the patterns observed in the present analysis are consistent with those national trends [3,14]. The lower SUID mortality observed among Hispanic infants is consistent with patterns reported in prior U.S. studies, where relatively favorable outcomes are noted despite socioeconomic disadvantage [3,12,14]. This has been described as the Hispanic Epidemiological Paradox and may reflect differences in social support, cultural practices, or health behaviors [10,15]. However, these mechanisms could not be examined in the current analysis due to the use of aggregated data.

National public health guidance in the United States has focused on reducing the risk of SUID through safe sleep recommendations and caregiver education. Public health campaigns such as the Back to Sleep initiative have emphasized placing infants on their backs to sleep and avoiding unsafe sleep environments. Reviews of these programs indicate that changes in infant sleep practices among caregivers and health professionals have been associated with reductions in sleep-related infant deaths in the United States [8,9]. Evidence-based safe sleep programs implemented in clinical and community settings have also been linked to lower rates of sleep-related infant deaths when caregivers receive consistent education and reinforcement of safe sleep practices [11]. Studies examining health care encounters before SUID indicate that opportunities often exist for clinicians to reinforce safe sleep counseling and caregiver education during routine health visits [4]. These recommendations highlight the role of preventive guidance during pregnancy and early infancy as part of broader strategies aimed at reducing SUID risk.

Medical professionals are central to the reduction of the incidence of SUID, notably through parental and caregiver education during routine clinical visits. Reports show that children who died from SUID had at least one recent encounter with their primary healthcare provider before death, indicating missed opportunities to provide protective or preventive strategies during these clinical visits [4]. Strengthening these encounters through standardized prompts within electronic medical records, alongside culturally tailored counseling approaches that address caregiver beliefs and practices, may improve the effectiveness of safe sleep education.

The United States guidelines equally highlight empirically supported infant safe sleep practices. Some recommendations from the American Pediatric Society include having infants sleep supine each time, ensuring the crib, bassinet, or cradle is firm and empty, discouraging the use of soft bedding, preventing overheating, making the environment smoke-free, and encouraging breastfeeding as a protective measure [23,24]. In addition, the use of pacifiers during sleep time has a protective effect against SUID [24]. According to these guidelines, infants less than six months old can share rooms with parents/caregivers. However, they should not share beds, and the health professional should begin teaching safe sleep practices during pregnancy and reiterate these strategies continuously after delivery [23].

Despite similarities in the American SUID preventive guidelines, they differ in their areas of focus. The recommendations emphasize public-health harm reduction strategies by encouraging physicians to advise against risky behaviors like smoking, alcohol, and drug use, bed sharing, as well as safety specifications for infant sleep products [23]. Conversely, the American guidelines encourage physicians and hospitals to practically demonstrate and educate parents/caregivers on safe infant sleep practices and the use of pacifiers as a preventive measure for SUID during routine antenatal and pediatric visits [24].

As a result, healthcare providers have ample opportunities to reinforce safe infant sleep practices from pregnancy through early infancy. During routine clinical visits, clinicians can reinforce and assess the infant’s sleeping environment and address concerns and misconceptions that could serve as important turning points in SUID prevention [3].

Studies in the United States have shown higher death rates from SUID among individuals of certain demographic populations, particularly those who are disadvantaged [3,14]. Interestingly, studies show a wide difference in SUID mortality rates among NHB and Alaskan Native infants compared to NHW infants, and these differences were influenced by various determinants of health [3]. Hence, these wellness and pediatric visits are important platforms for educating families, given the socioeconomic challenges that exist in the United States. Though several other factors, such as infant sleep practices and social determinants of health, can contribute to these disparities [14], the continuous teaching and reinforcement of SUID preventive measures during these visits can narrow the disparities associated with SUID.

Several mechanisms may help explain the patterns observed across maternal and infant characteristics. Low birth weight and early gestational age may reflect physiologic vulnerability during infancy. Infants born prematurely or with very low birth weight often have immature cardiorespiratory and neurological systems, which may influence arousal responses and respiratory regulation during sleep [5]. This concept aligns with the Triple Risk Model, which proposes that SUID occurs when a biologically vulnerable infant, particularly those born preterm or with low birth weight, encounters a critical developmental period in the presence of an external stressor such as unsafe sleep conditions [5,15]. Additionally, immature respiratory control in early infancy contributes to an unstable balance of breathing patterns, increasing susceptibility to apnea and consequent oxygen deprivation [5,15]. These physiological vulnerabilities alone do not invariably result in death; rather, they interact synergistically with extrinsic environmental conditions such as unsafe sleep positions and soft bedding. Maternal age and maternal education may also reflect differences in social conditions, health literacy, and access to health services during pregnancy and infancy. Research examining infant mortality in the United States has shown that maternal educational attainment is associated with infant survival and reflects broader socioeconomic conditions that shape maternal health and infant care practices [12]. Differences in infant mortality across racial and ethnic groups have also been documented in national studies and may reflect variation in social conditions, access to health services, and community-level factors affecting maternal and infant health [16]. Evidence from quality improvement initiatives in neonatal care has also shown that targeted education on safe sleep practices among Black infants can improve compliance with recommended sleep practices, suggesting that preventive education may influence risk-related behaviors in some settings [7].

Strengths and limitations of the study

The study has several strengths and limitations that should be considered when interpreting the findings. A major strength is the use of a large national dataset that captures births and infant deaths across the United States over a multi-year period, allowing examination of population-level patterns across multiple maternal and birth characteristics. The use of standardized national vital statistics data also supports consistency in the measurement of births and infant deaths.

However, several limitations should be acknowledged. The analysis relied on aggregated data derived from national vital statistics rather than individual-level records. As a result, detailed clinical or behavioral variables could not be examined. In addition, socioeconomic assessment was limited to maternal education, as other indicators, such as income or household characteristics, are not available within the aggregated CDC WONDER dataset. Information on sleep environment, parental behaviors, prenatal substance exposure, or caregiver awareness of safe sleep guidance was not available within the dataset, limiting the ability to identify specific modifiable risk factors or inform targeted interventions.

Furthermore, some variables used in national vital statistics records may contain missing or incomplete data. The study design is descriptive and cross-sectional; therefore, relationships between characteristics and mortality rates should be interpreted as associations rather than causal effects, and potential confounding factors could not be accounted for. While the findings highlight population-level disparities, they should not be interpreted as evidence of independent effects of individual variables. Future research using individual-level linked data may allow a more detailed assessment of clinical, behavioral, and environmental factors and support the development of more targeted prevention strategies.

## Conclusions

This study highlights differences in SUID across maternal and birth characteristics in the United States using national vital statistics data. Mortality rates varied across maternal age, maternal education, birth weight, gestational age, and maternal race and ethnicity. Higher rates were observed among infants born to younger mothers, mothers with lower educational attainment, and infants with lower birth weight or earlier gestational age. These findings support continued attention to social and clinical factors linked with infant health and early life conditions. Public health strategies that promote safe sleep practices and strengthen maternal and infant care remain important components of prevention efforts. Future research using individual-level data should examine additional clinical, behavioral, and environmental factors that may influence SUID risk and inform targeted prevention strategies.
